# Recognition of real and artificial intelligence-generated faces

**DOI:** 10.1177/03010066261442089

**Published:** 2026-04-29

**Authors:** Nicole Sulimani, Alexis van Hunenstijn, Andrea Albonico

**Affiliations:** 1 Department of Psychology, 1011 University of the Fraser Valley, Abbotsford, Canada

**Keywords:** Face recognition, artificial intelligence, response bias, covert recognition, generative AI

## Abstract

Artificial intelligence (AI) is changing society in major ways. AI can create content that entertains, but it can also mislead people. One part of this change is the ability to produce human-like AI content, including voices and faces. In psychology, AI-generated faces have started to be used to study face perception, thanks to their flexibility and convenience. This choice appears to be supported by recent evidence indicating that AI-generated faces are indistinguishable from human faces. However, other findings suggest that AI- or computer-generated faces may be evaluated differently than real faces, with AI-generated faces even leading to hyperrealism. To address this, we conducted a study to investigate whether human and AI-generated faces are recognized similarly in a memory task, and whether their recognition is related to our evaluation of these faces. Results show that recognition accuracy was significantly higher for AI faces than for real faces. Critically, classification (i.e., the ability to correctly classify a face as human or AI-generated) accuracy was significantly better than chance level for both real and AI-generated faces, with no difference between the two. These results suggest that humans can correctly discriminate AI-generated and human faces, and that AI-faces might be recognized better than human ones, suggesting that AI-generated faces might engage human perceptual and memory systems differently from real faces. However, recognition and classification accuracy were not significantly correlated, suggesting that participants might be partially unaware of their increased performance with AI-generated faces.

## Introduction

In recent years, artificial intelligence (AI) has advanced rapidly, transforming the way people interact with technology and bringing about social changes (Xie, 2023). For instance, companies like Apple have introduced their own AI system (Apple Intelligence), and many social media platforms, such as Facebook, have added AI-generated content. Due to the extreme popularity of these companies, most people are now regularly exposed to AI content. These changes are also visible in the scientific community, where publications of AI-related research papers have grown from less than 8,500 in 2000 to more than 57,000 in 2024.^1^ Although some progress has been made in developing automatic techniques that detect fake content (Agarwal et al., 2019; Li et al., 2020), current tools are not accurate enough to discriminate between real and fake content (Chaka, 2023): as a result, it has become increasingly difficult to distinguish between real and AI-generated material.

A specific case of this problem involves distinguishing between real and AI-generated faces. Indeed, AI-generated faces are now widely available and used for a variety of purposes, some of which, unfortunately, include spreading misinformation via fake social accounts (Hatmaker, 2020; Nazar & Bustam, 2020). Thus, it is critical to investigate whether humans can accurately discriminate between real and AI-generated faces. However, only a few researchers have explored this issue. Nightingale and Farid (2022) investigated how humans perceive and differentiate between real and AI-generated faces through a series of three separate experiments. In the first experiment, 315 participants were shown 128 faces that were either real or AI-generated, and the average classification accuracy was only 48.2%, which was not significantly different than chance. Interestingly, white faces yielded the lowest classification accuracy, with male white faces being misclassified more often than female white faces. The researchers theorized that these results were due to the AI software (StyleGAN2) being trained primarily on white male faces, making these generated faces more realistic. Their second experiment followed the same procedures but included trial-by-trial feedback (Nightingale & Farid, 2022). However, results replicated findings of the first experiment, suggesting that feedback did not improve accuracy. In their third experiment, they aimed to determine if AI-generated faces were perceived as more trustworthy than human ones. They asked 223 participants to rate the trustworthiness of 128 faces on a scale of 1 (very untrustworthy) to 7 (very trustworthy). Results showed that AI-generated faces were rated 7.7% more trustworthy than human faces.

**Figure 1. fig1-03010066261442089:**
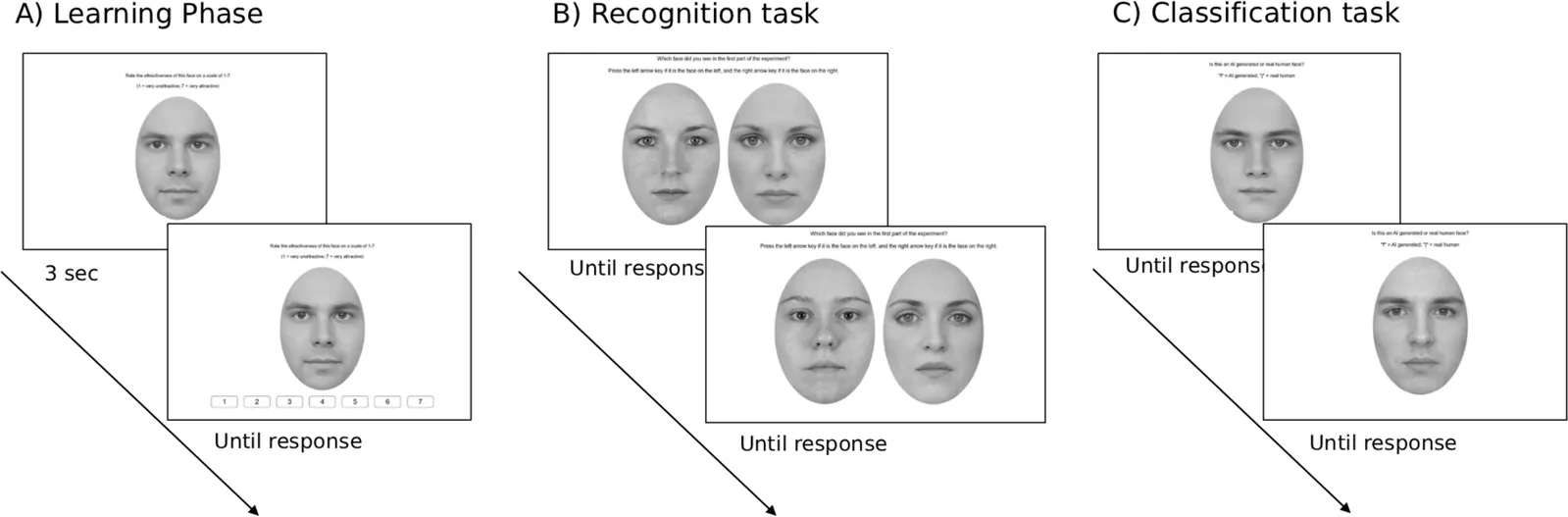
Examples of trials in the different phases of the experiment. (A) It shows one trial in the Learning Phase where participants were asked to rate the attractiveness of each face. (B) It shows two trials for the Recognition Task, in which participants were asked to indicate which one of the two faces on the screen they saw before; and (C) It shows two trials of the Phase 3 Classification Task, where participants were required to classify each face either as real human or AI-generated. Note that the sizes of the stimuli in the figure do not reflect the actual sizes used. AI=Artificial Intelligence.

Similar results were found by Shen et al. (2021): humans were unable to distinguish synthetic faces from real faces under different circumstances. This included displaying faces with the full context (i.e., hair, face contour, chin, etc.) and displaying only the face region under varied lighting conditions. Furthermore, two additional studies have suggested that AI-generated faces might not just be indistinguishable from actual human faces, but may be perceived as even more realistic-looking than real faces (Miller et al., 2023; Tucciarelli et al., 2022) – a phenomenon called *AI hyperrealism* (Miller et al., 2023). In line with the face-space theory, Miller et al. (2023) found that AI hyperrealism could be explained by the cumulative effects of different cues, such as AI faces being more average, familiar, attractive, and less memorable than human faces. Such characteristics might also explain why AI faces tend to be judged as more trustworthy than human faces (Nightingale & Farid, 2022) and why participants displayed increased social, conforming to faces perceived as real, independently of their actual realness (Tucciarelli et al., 2022).[Fig fig1-03010066261442089]

Thus, these findings suggest that humans cannot reliably discriminate between real and AI-generated faces, and that this might be due to some of the featural and surface cues that characterize AI-faces. The current study aims to investigate participants’ ability to recognize AI-generated faces versus human faces using a two-alternative forced-choice (2AFC) memory task – an indirect method not used in the previously mentioned studies. The 2AFC paradigm was chosen because previous evidence suggests that this task is more criterion-free than the Yes/No paradigm, as the decision is based on relative familiarity between the two options rather than an absolute, subjective threshold (Egan, 1975; Macmillan, 1991). Here, we were interested in testing whether performance in recognizing AI-faces is higher than that of real faces. Since AI-generated faces often display distinct characteristics (e.g., perfect symmetry), it is possible that recognition of AI-generated faces would be better than that of real human faces. On the contrary, if AI faces are recognized less accurately, it may indicate that their regularity and lack of distinctiveness could make their encoding and retrieval more difficult. We then also investigate whether the recognition performance with AI-generated and real faces correlates with the ability to discriminate between these two types of faces. If a difference in recognition accuracy is observed between AI-generated and human faces, it could suggest that the perceptual and encoding mechanisms engaged by AI faces are different from those used for real human faces.

## Methods

### Participants

Sixty participants were recruited and completed the test (29 females, 29 males and 2 others; mean age = 37.1 years, SD = 8.2 years, age range = 20–50 years old; 50 right-handed, 9 left-handed and one ambidextrous). Data from six participants were excluded due to excessive timeout or repetitive keys, resulting in a final sample of 54 participants for analysis (27 female, 25 male, 2 other; mean age = 37.0 years, s.d. = 8.0 years, age range = 21–50 years old; 46 right-handed and 8 left-handed). All were native English speakers, had normal or corrected-to-normal vision, no history of neurological or psychiatric conditions that could affect face perception, and all participants were reimbursed $2 for their time. The protocol was approved by the Institutional Review Boards of the University of the Fraser Valley, and all subjects gave informed consent in accordance with the principles of the Declaration of Helsinki.

### Stimuli

A total of 64 stimuli were used in the test: 32 real human faces and 32 AI-generated faces (half males and half females for both). Real human face stimuli were drawn from the Chicago Face Database (Ma et al., 2015), while AI-generated faces were sourced from Generated Photos (https://generated.photos), a website where one can generate customizable AI faces with the ability to change the sex, race, eye colour, hair colour, age, emotion, and more. To ensure that the real and AI images were comparable, each AI face was generated to closely match a specific real face on key visual attributes, including age range, eye and hair colour, and facial structure. We included equal numbers of male and female faces, all white, ranging in ages from approximately 20 s to late 30 s, with neutral expressions. To control for extraneous variables, all stimuli were edited to exclude hair, piercings, facial hair, glasses, or any other accessories. Additionally, each image was converted to greyscale and cropped using Adobe Photoshop CC 2014 (www.adobe.com) to include only the inner facial region, so that each image was placed within an oval aperture of 415 × 590 pixels. Removal of internal and external facial features was done to minimize reliance on image-based cues and ensure that recognition relied on face-specific mechanisms.

### Procedure

The tests were controlled by TESTABLE (www.testable.org). Participants were instructed to always conduct the experiment alone and in a quiet environment with the screen at an arm's length distance. The screen resolution was calibrated using a credit card as a standardized unit of measurement before the experiment began, to ensure consistency in stimulus size. The test consisted of three distinct phases (see Figure 1).

*Phase 1: Learning Phase:* The experiment began with a learning phase in which each participant was shown 32 faces: 16 *AI-generated* and 16 *real human*, all presented in a front-facing orientation. The full stimulus set consisted of 64 stimuli (32 AI-generated and 32 real human), which was divided into two equal halves. For each participant, one half served as the learning set and the other half served as the foil stimuli in the subsequent recognition task. The assignment of halves was counterbalanced so that the same stimuli acted as ‘target’ faces for half of the participants and as ‘foil’ faces for the other half. On each trial, a single face was presented, and participants rated its attractiveness on a 7-point Likert scale (1 = ‘less attractive’, 7 = ‘very attractive’), regardless of their sexual orientation. To ensure adequate encoding – given that this was an implicit learning phase, where participants were not informed that their memory would later be tested – each face remained on screen for three seconds before responses were permitted, with a maximum response window of 15 s. Participants provided their ratings by clicking the corresponding button with the mouse.

*Phase 2: Recognition Task:* Following the learning phase, participants completed a two-alternative forced-choice recognition task. In this phase, each participant was presented with 32 trials. On each trial, two faces were presented side by side, and participants had to indicate which face they had already seen. The size of these faces was reduced by 20% relative to the original face size during the learning phase to minimize low-level picture-matching strategies. Participants indicated which face they had seen before by pressing the left or right arrow key, corresponding to the face's position on the screen. The two faces remained on the screen until the participant's response or until a 10-s timeout. The position of the target face was counterbalanced across trials, and the trials were randomized across participants. Since there were 32 trials and 2 stimuli per trial, a total of 64 stimuli were used in this phase (32 AI-generated and 32 real human), and each target stimulus was shown to each participant only once. Trials were organized so that half of the trials featured an AI-generated target and the other half a real human target (the same as the ones shown in phase 1). The distractor faces were randomly chosen from the list of foil stimuli for each participant, so that each foil face appeared only once. Consequently, face pairings varied across participants, minimizing potential stimulus-pair confounds. The foil and target faces were always of the same gender. Furthermore, the foil and target faces could be of the same type (both *real human* or both *AI-generated*) or not (where either the foil is a *real human* face and the target is an *AI-generated* face, or vice versa), thus creating four combinations which appeared the same number of times.

*Phase 3: AI vs. Human Classification Task*: Next, participants completed a classification task. The same 32 target faces of the recognition task were presented individually, and participants were instructed to decide whether each face was AI-generated or human. Participants responded by pressing either ‘f’ or ‘j’, with key-category mappings counterbalanced across participants (i.e., for half of the sample ‘f’ indicated AI-generated and ‘j’ indicated human, and vice versa for the other half). Each stimulus remained on the screen until the participant's response or until a 10-s timeout. The trial order was randomized for each participant. After having classified all faces, participants provided a confidence rating indicating how certain they felt in distinguishing AI-generated from human faces (six-point scale ranging from ‘not confident at all’ to ‘extremely confident’). Finally, they completed an open-ended response describing the cues or strategies they used to make their judgments.

### Data Analysis

We divided our analysis into four main sections. First, we investigated whether AI or human faces were rated differently on attractiveness and whether such ratings were associated with any demographic variables of the participants. Second, we compared the recognition accuracy and response times of AI and human faces in the recognition task. Third, we investigated whether participants could correctly classify AI and human faces and identified the cues they used for such classification. Finally, we investigated which factors might have affected the participants’ performance in the recognition task, i.e., whether any correlation existed among the performance in the three phases.

## Results

**Figure 2.**
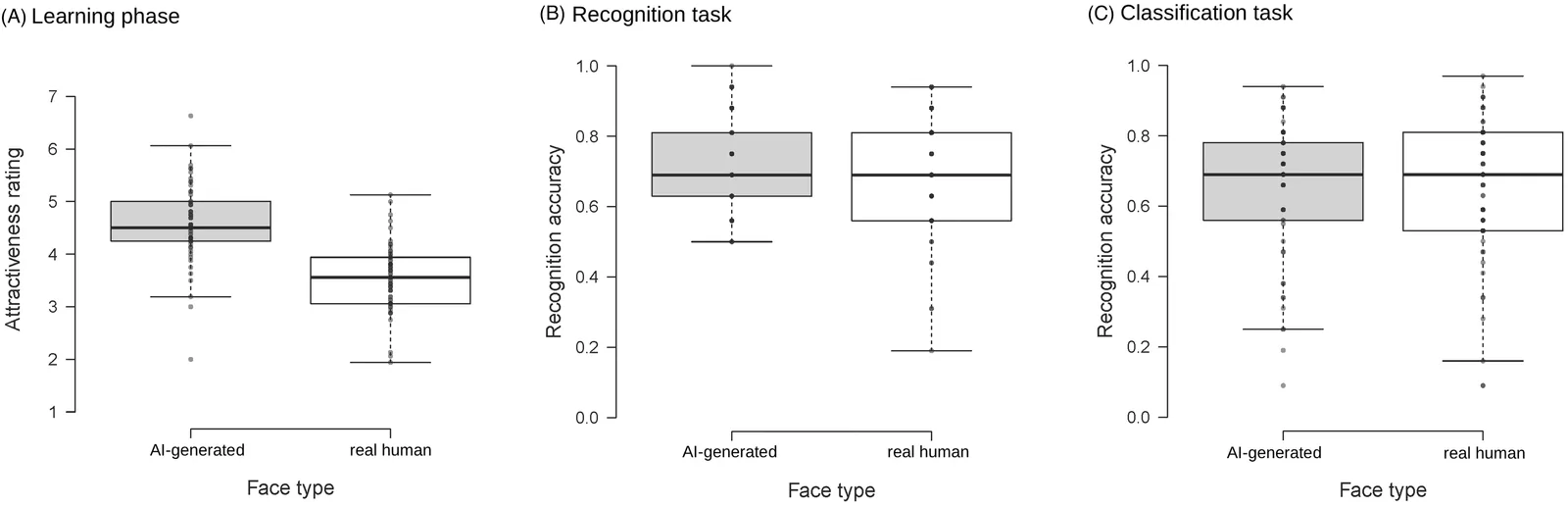
Difference in performance between AI-generated and real human faces in the three phases of the experiment. (A) It depicts the attractiveness ratings from the Learning Phase; (B) it depicts accuracy From the Recognition Task; and (C) it depicts accuracy from the Classification Task. AI=Artificial Intelligence.

*Phase 1: Learning Phase:* In the learning phase, AI-generated faces (M *=* 4.58, SE = 0.11) were rated significantly more attractive than human faces (M *=* 3.55, SE = 0.09; *t*(53) = 9.59, *p* < .001, *d* *=* 1.31; Figure 2A). Participants also rated female faces (M = 4.42, SE = 0.09) as significantly more attractive than male faces (M = 3.71, SE = 0.09; *t*(53) = 9.88, *p* < .001, *d* = 1.35). Since both the type of face and the face gender influenced the attractiveness ratings, we also conducted a repeated-measure ANOVA with face type (real human vs. AI-generated) and face gender (male vs. female) as within-subject factors. Such analysis confirmed the significant main effects of face type (F(1,53) = 91.9, p < .001, $\eta_{p}^{2}\;\;$ 0.634) and of face gender (F(1,53) = 97.7, p < .001, $\eta_{p}^{2}\;\;$ 0.648), as described above, but the interaction between the two effects was not significant (F(1,53) = 0.5, p = .462, $\eta_{p}^{2}\;\;$ 0.010). Demographic variables, such as age, gender, sex, and ethnicity, were not significantly associated with the attractiveness ratings. These results suggest that AI-generated faces are found to be more aesthetically appealing.

*Phase 2: Recognition Task:* In the recognition task, we aimed to answer our main research question – *are AI and human faces recognized differently?* Here, accuracy was significantly higher for AI-generated faces (M = 72.9%, SE = 2%) than for real faces (M = 66.6%, SE = 2%; *t(53)* *=* *-*2.59, *p* = .012, *d* = 0.35; Figure 2B). Critically, recognition accuracy was significantly higher than chance for both real (*t*(53) = 7.6, p < .001) and AI-generated faces (*t*(53) = 13.3, p < .001). On the other hand, there was no significant difference in response times between the two conditions (real faces: M = 2451 ms, SE = 124 ms; AI-generated: M = 2547 ms, SE = 105 ms; *t*(53) = 1.181, p = .243, *d* = 0.16). Since in the recognition test, the two stimuli on each trial could be a mix of real and AI-generated faces, we also conducted a repeated-measure ANOVA to investigate if the face pairings influenced the performance in the recognition task. We found a significant effect of the face pairing (F(2,106) = 5.1, p = .008; $\eta_{p}^{2}\;\;$ 0.087). Post hoc analyses (dependent t-tests with Bonferroni correction) revealed that recognition accuracy was lower when both stimuli on the faces were AI (M = 65.5%, SE = 2%) compared to when both stimuli were real faces (M = 74.8%, SE = 2%; p = .016), but not to when the screen included a mix of real and AI faces (M = 69.0%, SE = 2%; p = .822). The difference between the two real faces and a mix was not significant either (p = .061). Since in the mixed condition (i.e., one AI-generated and one human face) the target face could either be AI-generated or human, we also conducted an additional dependent t-test to investigate if the type of target face affected performance in the mixed condition: we found a significantly higher accuracy in recognizing AI-generated faces (M = 79.8%, SE = 2%) compared to human faces (M = 58.2%, SE = 3%; t(53) = 5.6, p < .001, d = 1.10) in the mixed condition. The effect of the face pairing was significant also on the response times (F(2,106) = 9.5, p < .001, $\eta_{p}^{2}\;\;$ 0.151): participants were slowest when both faces on the screen were both AI-generated (M = 2738 ms, SE = 136 ms) compared to both when the two stimuli were both real faces (M = 2442 ms, SE = 111 ms; p = .014) and when the two faces were a mix (M = 2401, SE = 105 ms; p = .001). An additional dependent t-test for the mixed condition revealed that response times for trials where the human face was the target one (M = 2454 ms, SE = 146 ms) were not significantly different from trials were the AI-generated face was the target one (M = 2348 ms, SE = 83 ms; t(53) = -0.96, p = .343, d = -0.121). These results suggest that participants had more difficulty recognizing faces when shown both AI stimuli.

*Phase 3: AI vs. Human Classification Task*: In the AI vs. Human classification task, no differences were found in accuracy between AI (M = 64.9%, SE = 2%) and real faces (M = 64.7%, SE = 3%; *t*(53) = 0.05, *p* = .964, d = 0.006; Figure 2C). Critically, classification accuracy was better than chance level for both human (t(53) = 5.1, p < .001) and AI-generated faces (t(53) = 5.4, p < .001). However, participants’ reaction times were significantly faster for AI faces (M = 1600 ms, SE = 80 ms) compared to real faces (M = 1707 m; SE = 106 ms; t(53) = -2.36, p = .022, d = -0.32). Most participants reported moderate confidence in the classifications (M = 3.52, SE = 0.16), and the confidence ratings were not significantly correlated with either the accuracy (r = -0.46, n = 54, p = .740) or the response times (r = 0.177, n = 54, p = .201) in the classification task. For this task, we also calculated participants’ response bias, *c* (Macmillan & Creelman, 1990): hits were defined as correctly identifying a face as AI-generated, and false alarms were defined as incorrectly identifying a real face as AI-generated. Thus, a positive value for *c* indicates a tendency to classify faces as real, while a negative value for *c* indicates a tendency to classify faces as AI-generated. The average criterion for our participants was 0.003 (s.d. = 0.374), which didn’t significantly differ from a neutral bias of *c* = 0 (t(53) = 0.0635, p = .9496). This result shows that our participant didn’t have a bias in the classification task. Qualitative analyses of open-ended responses showed that participants relied most frequently on texture and surface cues (e.g., skin smoothness, blemishes; 67% of the participants), followed by affective/intuitive cues (e.g., using ‘gut feeling’ or emotional reaction; 44%), then featural cues (e.g., looking at specific parts of the face, such as eyebrows, eye shape; 37%) and configurable cues (e.g., symmetry, spacing; 35%). Participants relied least on image quality/realism cues (e.g., photo sharpness, how real/fake the image appears; 19%). Note that some participants’ responses included multiple cues and therefore the reported percentages reflect the proportion of participants mentioning each category rather than summing to 100%.

**Figure 3.**
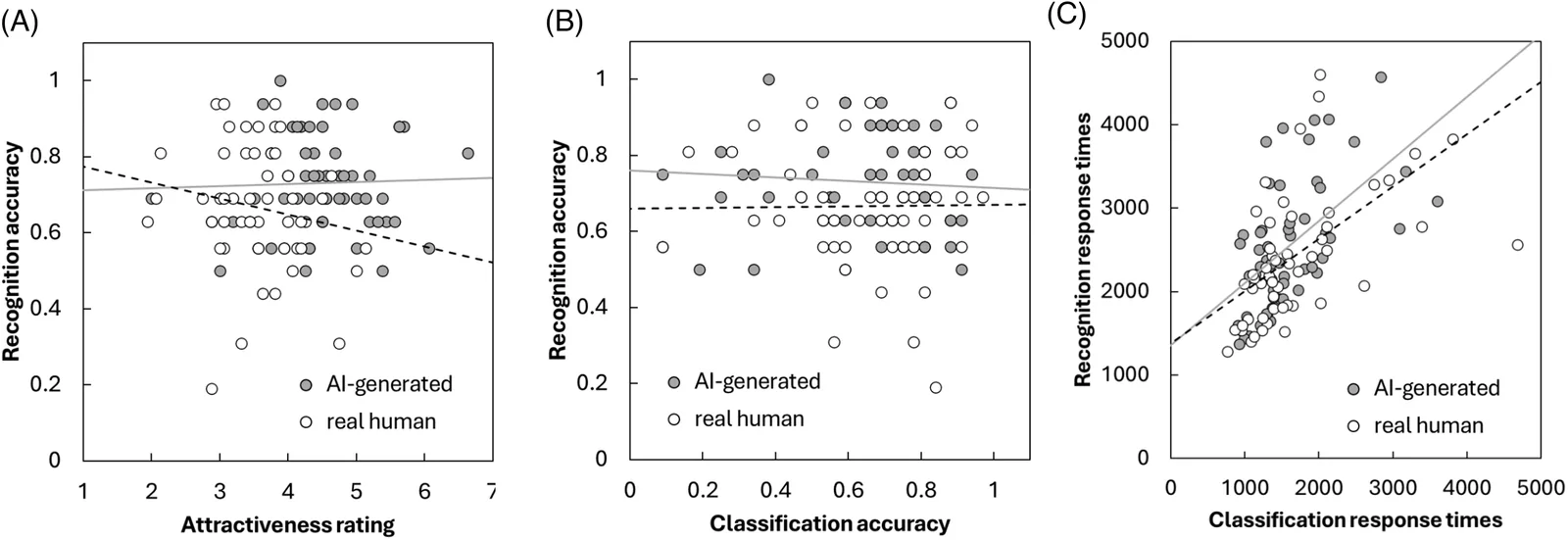
Correlation among the performance in the three different tasks, separately for AI-generated and real human faces. (A) It depicts the correlation between accuracy in the Recognition Task and the attractiveness ratings from the Learning Phase; (B) it depicts the correlation between the accuracy in the Recognition and Classification Tasks; and (C) it depicts the correlation between the response times in the Recognition and Classification Tasks. The grey lines depict the regression lines for the AI-generated faces, while the black dashed lines depict the regression lines for the real human faces. AI=Artificial Intelligence.

*Relationship among the different tasks:* Lastly, we investigated whether performance in the recognition test was influenced by performance in the other two tasks. No significant correlations were found between the overall rating in the attractiveness judgement and either the accuracy (r = -0.060, n = 54, p = .669) or response times in the recognition task (r = -0.075, n = 54, p = .589). The same pattern of results was found when correlations were run separately for AI-generated and real human faces (all ps > .05; Figure 3A). Performance in the recognition task also wasn’t affected by the performance in the classification task: indeed, the correlation between accuracy in the recognition and classification tasks (r = -0.075, n = 54, p = .590), as well as the correlation between the accuracy in the recognition task and the confidence ratings in the classification task (r = 0.234, n = 54, p = .088) were not significant. However, the correlation between the response times in the two tasks was significant (r = 0.256, n = 54, p < .001). The same pattern of results was found when correlations were run separately for AI-generated and real human faces (Figure 3B).

To assess whether stimulus properties influenced performance independently of participant-level differences, we conducted stimulus-level analyses. A stimulus-level Pearson correlation analysis was conducted to examine whether mean attractiveness ratings predicted performance in the recognition and identification tasks. Attractiveness was negatively associated with recognition accuracy in Phase 2 (r = −0.41, n = 64, p < .001), indicating that more attractive faces were less likely to be correctly classified as familiar or unfamiliar. In contrast, attractiveness was not related to identification accuracy in Phase 3 (r = −0.05, n = 64, p = .71), when participants judged whether faces were AI-generated or real.

## Discussion

Regarding our primary hypothesis, our results show that AI-generated faces are recognized better in a memory recognition task, compared to human faces. AI-generated faces were also judged as being more attractive than human faces. Critically, our participants were able to classify human and AI-generated faces above-chance levels. Correlational analyses revealed that performance in the different tasks was mostly independent. A few results deserve some comments.

First, in our learning phase, we found that AI-generated faces were judged as being more attractive than real faces. This result is in line with previous reports finding that AI-generated faces are judged as being more trustworthy (Nightingale & Farid, 2022) and overall, as more attractive than human faces (Miller et al., 2023). Opposite results were, however, found in another study in which computer-generated faces were rated as less attractive than real faces, regardless of the participants’ familiarity with computer-generated stimuli (Di Natale et al., 2024). The discrepancy between these different results might be linked to the different degrees of realism of computer-generated and AI-generated faces. Indeed, some studies have found a positive correlation between face evaluations and realism, with more realism leading to more favourable ratings (Cheetham et al., 2014; Kätsyri et al., 2015). One possible reason why AI-generated faces are judged more favourably, either in trustworthiness or attractiveness, might be because synthesized faces tend to look more like average faces (Rubenstein et al., 2002; Sofer et al., 2015). Indeed, it has been proposed that generative adversarial networks (GANs) are biased toward the statistical regularities of their most common inputs (Karras et al., 2019), thus making AI faces appear more average than their human counterparts (Miller et al., 2023). A useful framework here is the face-space theory (Valentine, 1991; Valentine et al., 2016), a hypothetical multidimensional space in which faces are coded along dimensions according to how much they differ from an average face located at the centre. Human faces are expected to be normally distributed within this space, whereas AI faces tend to cluster more around the average dimensions due to the bias toward average features that generative algorithms are trained on (Karras et al., 2019; Miller et al., 2023).

Second, we found that overall, AI faces were recognized better than human faces in the memory recognition task. Previous studies investigating AI stimuli (Miller et al., 2023; Nightingale & Farid, 2022; Shen et al., 2021; Tucciarelli et al., 2022) did not test whether AI faces are recognized differently from human ones. Therefore, our study is the first to demonstrate this. However, a previous study (Di Natale et al., 2024) tested the recognition accuracy of computer-generated faces in a memory task by using two variants of computer-generated faces: computer-generated faces with low realism (CG-low) and computer-generated faces with high realism (CG-high) – where realism was manipulated by altering the texture resolution of the faces. Their results show that computer-generated faces, especially those less realistic, were less accurately recognized than human faces; however, this difference disappeared in participants with great exposure to digital characters and stimuli. They concluded that computer-generated faces are less efficiently discriminated compared to real faces due to their lack of surface cues – such as pigmentation and reflectance – that ultimately help individuals recognize faces. Moreover, they believed that computer-generated faces are harder to discriminate due to their lack of realism, which results in the faces looking too similar. The increased performance in participants who are regularly exposed to computer-generated content would be explained by perceptual narrowing (Di Natale et al., 2024). Such interpretation seems to be in accordance with the face-space theory (Valentine, 1991; Valentine et al., 2016), where faces that are closer to each other (and to the average) become less memorable and therefore more difficult to recognize. If that is the case, why did we find better accuracy for AI faces? Miller et al. (2023) proposed that the same perceptual qualities of faces that contribute to the averageness of a face also contribute to the hyperrealism phenomenon: AI-generated faces might look more average than human faces without losing the facial characteristics and/or cues that make a face memorable and recognizable. Our results also show that face pairing influenced performance in the recognition task. Indeed, accuracy was higher when the two stimuli on the screen were a mix of AI and human faces (particularly when the AI face was the target face), and lower when they were both AI faces. This might be due to the greater homogeneity of AI-generated faces, as AI faces. A likely explanation of this result is the greater homogeneity of AI-generated faces, which tend to appear more average than their human counterparts (Miller et al., 2023) and are all closer to the average of the face space, thus making the recognition more difficult – a result not too dissimilar from what was found by (Di Natale et al., 2024). The increased homogeneity of AI faces might also explain performance in the mixed trials: because AI faces tend to look more similar to each other, participants may have been more prone to confuse a novel AI foil with a previously seen AI target. This would decrease accuracy when the real face was the correct choice and, conversely, increase performance when the AI face was the target. These results suggest that the enhanced recognition of AI-generated faces might also be partially dependent on the pairing condition in the alternative forced-choice task. So, future studies could examine recognition using single-stimulus (Yes/No) designs to determine whether the effect generalizes to other tasks. A further interesting question that future studies should try to answer is whether the different recognition accuracy (and position in the face space) of AI and real faces also reflects different perceptual and encoding mechanisms engaged by the two types of faces.

Third, we found that both AI-generated and human faces were correctly classified above-chance level, with no significant difference between them. Classification accuracy in our study is higher compared to the previous reports (Miller et al., 2023; Nightingale & Farid, 2022; Shen et al., 2021; Tucciarelli et al., 2022) and our is the first report in which participants were able to distinguish AI from human faces above-chance level (even if still distant from an ideal performance, since both classification accuracies were around 65%). Indeed, previous reports have found either that people cannot classify AI and human faces better than chance level (Nightingale & Farid, 2022) or that people may over-identify AI faces as human (Miller et al., 2023; Shen et al., 2021; Tucciarelli et al., 2022), a phenomenon called *AI hyperrealism*. These studies showed that the realism of synthetic faces can extend across race and gender, and that AI stimuli might have intrinsic features that lead them to be perceived as more real (Tucciarelli et al., 2022). Miller et al. (2023) suggested that the increased likelihood of judging AI faces as human can be explained by the cumulative effects of different cues, such as AI faces being more average, familiar and attractive than human faces. So, why were our participants able to correctly classify AI and human faces? One possible explanation is that, while all the studies mentioned above used the StyleGAN2 algorithm to create AI faces, we used a more available and easily accessible website (https://generated.photos) to create our AI faces, raising the possibility that our stimuli might have included some cues that could have helped in the classification task. Another possible explanation is that, while in the works cited above, participants were only exposed to the AI stimuli and asked to classify them, in our study, the classification task was the last phase of the experiment: the increased familiarity with the AI stimuli (participants would be exposed to the AI stimuli one time in the learning phase, a second time in the recognition task, and a last time in the classification task) might have disclosed some cues for correctly classifying the faces as AI or human. This result seems to be in accordance with previous findings showing that participants’ perceptions of credibility of AI material (faces and voices) were influenced by their prior knowledge of the AI-generated media and their familiarity with the source material (Gregory & Monteiro, 2023). Interestingly, we found that confidence ratings were not significantly correlated with accuracy in the classification task, suggesting that participants might not be aware of the validity of the cues they used to classify AI and human faces. A similar result was found by (Miller et al., 2023), who found that higher errors for AI faces were associated with high confidence levels, i.e., participants who made the most errors in classifying AI faces were the most confident in their judgments. One final factor that could have affected our results in the recognition and classification task is the fact that the same face images were used between the learning and recognition/classification phases (even if in the recognition phase, face images were 20% smaller since two faces at a time were presented on the screen). This was done because the AI face-generation software we used could not accurately generate multiple images of the same face without altering some of its features. The use of the same images leaves open the possibility that participants used low-level picture-matching strategies to complete the tasks, which may have contributed to the above-chance performance we observed. Thus, future studies should replicate our findings by using different facial images between the learning and recognition/classification phases to ensure that results can be attributed to face-specific mechanisms. Similarly, in the present study, we have eliminated all external facial features, such as hair and the face contour, to ensure that recognition relied on face-specific mechanisms. However, future studies should also investigate whether recognition and classification of AI-generated faces differ when such external features are included. This might be particularly true for the face contour line, which is undoubtedly a determinant of facial structure and can affect recognition performance (Latif & Moulson, 2021).

In order to better understand our participants’ judgements, open-ended responses were examined to identify the facial cues that they used to classify faces as human or AI. Each participant's response was categorized into one or more facial cue types according to the strategies they described using to make their judgments. In comparison, Miller et al. (2023) observed that participants primarily used featural cues in their judgements. Although they argued that the participants misinterpreted these features – i.e., they use them in a way that reinforces hyperrealism. In other words, participants focused on cues that make AI faces seem more realistic rather than using cues that would help them correctly identify them as AI. The majority of our participants reported using texture/surface cues since AI-generated faces commonly lack imperfections, such as blemishes or rough skin. Very few participants noted relying on featural cues, such as examining the eyes, or configural/holistic cues, such as symmetry and the overall balance of the face. These differences suggest that, unlike in Miller et al. (2023) study where participants misunderstood potentially useful cues, our participants relied on different cues which might explain the better classification performance in our study. Our results are similar to those Gregory and Monteiro (2023) where participants were able to identify real and AI faces based on their relative unattractiveness, suggesting that aesthetic cues and implicit expectations play a significant role in classification judgments. Taken together, these results suggest that participants use different strategies and cues depending on the stimulus that needs to be classified.

Finally, we investigated whether performance in the recognition task was affected by performance in the other phases of the experiment (i.e., the attractiveness ratings and/or the classification task). At the participant level, we found no significant correlations between the accuracy in the recognition task and the attractiveness ratings or the accuracy (or confidence ratings) in the classification task. Only response times in the recognition and classification tasks were significantly correlated; however, given the overall pattern of results, it seems that these correlations are likely due to more general response factors. At the stimulus level, we found that attractiveness was negatively associated with recognition accuracy, indicating that more attractive faces were less likely to be correctly identified as familiar or unfamiliar. Conversely, in the classification task, attractiveness did not predict overall accuracy.

In conclusion, the present findings demonstrate that individuals perceive AI-generated faces differently compared to real human faces. More specifically, we found new evidence that AI-generated faces are recognized more accurately and judged more attractively than human faces, particularly when AI faces were to be compared with real ones. However, our correlational analysis seems to suggest that the increased memory for AI-generated faces might be partially indirect, since it doesn’t seem to depend on the participant's ability to discriminate AI-generated and human faces, nor their confidence level in this discrimination. Our findings also contribute to previous research by demonstrating that participants can classify AI and human faces above-chance levels, whereas other researchers only reported near-chance performance. These results suggest that AI-generated faces might engage human perceptual and memory systems differently from real faces. Understanding how people perceive and remember AI-generated content is important since AI has become increasingly ingrained in social media platforms, especially as some content is made to deceive viewers.

## Notes

1. Data from https://www.science.org/content/article/china-tops-world-artificial-intelligence-publications-database-analysis-reveals
